# Technical Outcome, Clinical Success, and Complications of Low-Milliampere Computed Tomography Fluoroscopy-Guided Drainage of Lymphoceles Following Radical Prostatectomy with Pelvic Lymph Node Dissection

**DOI:** 10.3390/diagnostics12102394

**Published:** 2022-10-01

**Authors:** Melvin D’Anastasi, Simone Ebenberger, Abdulmajeed Alghamdi, Andreas Helck, Annika Herlemann, Christian Stief, Wael Khoder, Christoph G. Trumm, Robert Stahl

**Affiliations:** 1 Medical Imaging Department, Mater Dei Hospital, University of Malta, MSD 2090 Msida, Malta; 2 Department of Radiology, University Hospital, LMU Munich, Marchioninistrasse 15, 81377 Munich, Germany; 3 Department of Urology, Albaha University, Albaha 65779-7738, Saudi Arabia; 4 Department of Urology, University Hospital, LMU Munich, Marchioninistrasse 15, 81377 Munich, Germany; 5 Radiology and Neuroradiology, Hirslanden Klinik Im Park, Seestrasse 220, 8027 Zürich, Switzerland; 6 Department of Urology, Helios-Amper Klinikum Dachau, Krankenhausstraße 15, 85221 Dachau, Germany; 7 Institute for Diagnostic and Interventional Neuroradiology, University Hospital, LMU Munich, Campus Grosshadern, Marchioninistrasse 15, 81377 Munich, Germany

**Keywords:** Multidetector Computed Tomography, lymphocele, prostatectomy, drainage, radiology, interventional

## Abstract

To evaluate the technical outcome, clinical success, and safety of low-milliampere CT fluoroscopy (CTF)-guided percutaneous drain (PD) placement in patients with lymphoceles following radical prostatectomy (RP) with pelvic lymph node dissection (LND). This retrospective analysis comprised 65 patients with PD placement in lymphoceles following RP under low-milliampere CTF guidance. Technical and clinical success were evaluated. Complications within a 30-day time interval associated with CTF-guided PD placement were classified according to SIR. Patient radiation exposure was quantified using dose-length products (DLP) of the pre-interventional planning CT scan (DLP_pre_), of the sum of intra-interventional CT fluoroscopic acquisitions (DLP_intra_) and of the post-interventional control CT scan (DLP_post_). Eighty-nine lymphoceles were detected. Seventy-seven CT-guided interventions were performed, with a total of 92 inserted drains. CTF-guided lymphocele drainage was technically successful in 100% of cases. For all symptomatic patients, improvement in symptoms was reported within 48 h after intervention. Time course of C-reactive protein and Leucocytes within 30 days revealed a statistically significant (*p* < 0.0001) decrease. Median DLP_pre_, DLP_intra_ and DLP_post_ were 431 mGy*cm, 45 mGy*cm and 303 mGy*cm, respectively. Only one minor complication (self-resolving haematoma over the bladder dome; SIR Grade 2) was observed. Low-milliampere CTF-guided drainage is a safe treatment option in patients with lymphoceles following RP with pelvic LND characterized by high technical and good clinical success rates, which provides rapid symptom relief and serves as definite treatment or as a bridging therapy prior to laparoscopic marsupialisation.

## 1. Introduction

Lymphoceles are collections of leaked lymphatic fluid with no distinct epithelial lining [1,2,3,4]. They commonly occur after surgical procedures which involve resection in areas with extensive lymphatic networks, causing disruption of lymph vessels, such as following radical prostatectomy (RP), perineal resections for genitourinary malignancies, vascular bypass surgery, renal transplantation and axillary lymph node dissection (LND) [1,4,5,6]. Lymphoceles are the most frequent non-functional complication of (RP) with pelvic lymph node dissection and were identified in up to 26% of patients post-RP in a study by Khoder et al. [7,8]. In the same study, 2.4% of patients post-RP had symptomatic lymphoceles and 1.9% required intervention [7,8]. Lymphoceles are commonly reported following both open retropubic radical prostatectomy (RRP) and robot-assisted radical prostatectomy (RARP) [9]. A recent study by Magistro et al. showed that, in addition to an extended lymph node yield, high-grade disease is associated with a higher risk in developing symptomatic lymphoceles, irrespective of the surgical approach [9]. Lymphoceles may be associated with morbidity due to compression of neighbouring structures or secondary infection [10]. They may cause deep-vein thrombosis (DVT) or even pulmonary embolism (PE) secondary to compression of pelvic veins [8,10,11].

There is no consensus on the optimal treatment for post-operative lymphoceles after RP [1]. Treatment options include percutaneous fine-needle aspiration, percutaneous catheter drainage, sclerotherapy, embolization during lymphangiography, and open or laparoscopic surgical evacuation with marsupialization [1,4,5,6,7,12,13]. Percutaneous catheter drainage +/− instillation of a sclerosing agent and laparoscopic fenestration are the most commonly performed therapeutic procedures. Laparoscopic fenestration has a high success rate of over 90% according to a number of studies [7,11,14] and is considered the surgical treatment of choice. Less invasive treatments such as imaging-guided PD, while having a lower success rate than laparoscopic marsupialisation, have an important role to play. These can be performed quickly, providing immediate decompression of large lymphoceles, i.e., lymphoceles compressing the pelvic veins, as well as superinfected lymphoceles. This enables rapid relief of symptoms in symptomatic patients. PD may obviate the need for surgery if no recurrence occurs following complete drainage or can serve as a bridging therapy until surgery can be performed. 

Percutaneous catheter drainage may be performed under Ultrasound (US) guidance or under Computed Tomography (CT) guidance. The latter may be performed using CT fluoroscopy (CTF) guidance or sequential CT guidance. CT-guided drainage provides advantages over US-guided drainage, particularly in overweight or obese patients with better visualisation of the lymphoceles and high-risk structures. The advantages of US-guided drainage over CT-guided drainage include the better availability of US and the possibility to perform drainage as a bedside procedure and at a reduced procedure cost with no radiation dose to the patient or interventional radiologist. CT-fluoroscopy guidance enables direct visualisation of the drain during insertion with a lower radiation dose to the patient compared to performing sequential CT scans, particularly when this is done using the low-milliampere technique.

The aim of our study was to evaluate the technical outcome, clinical success, and safety of low-milliampere Computed Tomography fluoroscopy (CTF)-guided percutaneous drain (PD) placement in patients with lymphoceles following RP with pelvic lymph node dissection.

## 2. Materials and Methods

Our study is a retrospective study of all consecutive patients who underwent PD placement in lymphoceles following RP with pelvic LND under low-milliampere CTF-guidance at our institution from May 2006 to August 2015. 

Our study was approved by the ethics committee of Ludwig-Maximilians-Universität München (LMU) (Project number: 518-16). Peri-interventional imaging studies and clinical patient charts were retrospectively reviewed. The principles of the Declaration of Helsinki were followed. Informed consent by the patient to undergo CTF-guided PD placement was obtained 24 h prior to, as well as directly before, the intervention.

### 2.1. Study Subjects

For each patient, the indication for CTF-guided PD placement had been discussed and confirmed by urologists and interventional radiologists in a multidisciplinary setting, based on clinical symptoms, laboratory parameters and abdominal ultrasound or CT examinations performed in inpatients or outpatients in the postoperative time interval. The clinical charts of 74 male patients referred to our department for PD placement in postoperative lymphoceles after RP with pelvic LND were retrospectively reviewed. Out of a total of 74 male patients following RP with lymphoceles referred for percutaneous CT-guided drainage, the following were excluded from further analysis: Five patients with a diagnosis of an abscess made after aspiration/drainage, two patients for whom CT images were no longer available and another two patients who only underwent aspiration but no drainage.

### 2.2. Peri-Interventional Imaging and Image Guidance

Prior to CT fluoroscopy-guided PD placement, contrast-enhanced cross-sectional images obtained within ≤48 h for each patient, such as CT, MRI or PET-CT, were reviewed by an interventional radiologist with >10 years of experience. 

All procedures were performed using one of the following CT scanners: Somatom Sensation 16, Somatom Definition AS+ or Edge (Siemens Healthineers, Erlangen, Germany) with CT fluoroscopy (CARE Vision CT^®^) capability. An unenhanced pre- and postinterventional CT scan of the abdomen was performed. For PD insertion trajectory planning, the pre-interventional CT scan included 5 mm slices, and coronal and sagittal reconstructions.

PD placement was carried out under intermittent quick-check CT fluoroscopic acquisitions, using low-milliampere CTF at a tube current-exposure time product of 10–30 mAs [15]. Standard radiation protection precautions for the operator during CTF included aprons, thyroid shields and protective eyewear (glasses) of 0.5 mm lead equivalent. Prior to sterile draping, an additional shield was applied onto the lower half of the patient to diminish scattered radiation. During CTF, angular beam modulation (Hand Care^®^) was activated, i.e., radiation exposure is switched off between a ten and two o’clock position of the X-ray tube to protect the operator’s hand.

A contrast-enhanced or unenhanced CT scan with multiplanar reconstructions was performed following PD placement to assess for possible peri-interventional complications.

### 2.3. Procedure

All procedures were performed by interventional radiologists with a minimum of 10 years of experience in CT-guided interventions. In patients with cardiorespiratory comorbidities, monitoring with pulse oximetry was performed during the intervention. Following sterile draping and disinfection of the skin overlying the planned PD entry site, local anesthesia with 10 to 20 mL of 2% Mepivacainhydrochloride (Scandicain^®^, AstraZeneca GmbH, Wedel, Germany) was applied. A small skin incision was performed and the PD (1. FleximaTM All Purpose Drain (APD), Boston Scientific Corporation, Marlborough, MA, USA or 2. ReSolve^®^ Non-Locking Drain Catheter, Merit Medical, South Jordan, UT, USA) was then introduced and advanced to the lymphocele using the curved trocar technique under intermittent quick-check CTF [16]. CTF was generally performed in the low-milliampere technique with tube current-time product values ranging between 10 and 30 mAs.

Following PD placement within the lymphocele, a non-contrast CT scan covering at least 10 cm above and below the entry point along the z-axis was performed to confirm that the final PD positioning was correct and exclude immediate complications. The PD was then fixed to the skin with a suture and covered with a sterile bandage. All patients were then transferred once again to the urology ward for clinical monitoring for 24 h. 

### 2.4. Assessment of Technical and Clinical Outcome and Complications

In a retrospective analysis of patients’ imaging studies available in the local picture archiving and communication system (PACS), radiology reports and other medical records, two experienced interventional radiologists (S.E.; M.D.; C.G.T.) evaluated the following: number of interventions, interventional technique (Trocar technique vs. Seldinger technique), number and size of drainage catheters, lymphocele location and longest diameter of the lymphoceles (in axial or coronal plane). Lymphoceles were classified as different types according to the classification by Khoder et al. [7] in consensus between a radiologist and urologist (M.D., W.K.). 

The technical and clinical success, as well as complications associated with CTF-guided PD placement, were evaluated during a post-interventional period of 30 days. The length of hospital stay for each patient and date and type of fenestration surgery (if performed) were recorded.

Technical success was defined as PD placement within the lymphocele with consecutive complete or near-complete fluid aspiration, yielding clear/yellow-coloured fluid or darker fluid if infected or haemorrhagic, and connection to a two/three-way stopcock and drainage bag. Any cases during which the PD could not be inserted into the lymphocele or aspiration was not possible were regarded as technical failure. 

Clinical success was defined as an improvement in symptoms within the first 48 h if symptomatic and normalization or marked improvement of inflammatory parameters (leucocytes, CRP) within 30 days after the intervention. Complications were evaluated according to the Cardiovascular and Interventional Radiological Society of Europe (CIRSE) [17].

### 2.5. Assessment of Patient Radiation Dose

CT dosimetry for all procedures was performed using the dose-length product (DLP), documented by the CT unit as primary dosimetric quantity data, according to Kloeckner et al. [18]. The DLP is defined as the dose in one CT rotation multiplied by the exposure length in mGy*cm. The DLP of the pre-interventional planning CT scan (DLP_pre_), the DLP of the sum of all intra-interventional CT fluoroscopic acquisitions (DLP_intra_) and the DLP of the post-interventional control CT scan (DLP_post_) were evaluated.

### 2.6. Statistical Analysis

Data analysis for this study was performed with R (R Core Team (2020). R: A language and environment for statistical computing. R Foundation for Statistical Computing, Vienna, Austria. URL https://www.R-project.org/, accessed on 22 June 2020, version 4.0.2). Discrete and continuous data were first assessed for normality using the Shapiro–Wilk test. Normally distributed variables are shown as mean ± standard deviation (SD). Variables which do not follow normal distribution are provided as median (25%-; 75%-quartiles). To examine the course of the blood parameters CRP and leucocytes over time in the 30-day post-interventional period, the values were log-transformed to achieve normal distribution. Generalized linear mixed models (GLMM) were applied. Fixed effects were given by the number of days after the intervention. Random intercepts were given by subject ID repeated by days. A level of significance of α = 0.05 was used in this study.

## 3. Results

Sixty-five male patients with lymphoceles following RP with pelvic LND could be included in our study.

### 3.1. Patient Characteristics

The mean patient age was 66 ± 7 years (range: 44–78 years). Fifty-two out of 65 (80%) patients had undergone an open prostatectomy with pelvic LND, while 13/65 (20%) patients had undergone a robot-assisted prostatectomy with pelvic LND. In addition, 63/65 (96.9%) patients presented with primary lymphoceles following RP, whereas 2/65 (3%) of patients had a lymphocele recurrence following laparoscopic marsupialization. The median time interval between surgery and intervention was 36 days (25%-; 75%-quartiles-17–70); 41/65 (63.1%) patients presented with one lymphocele, while 24/65 (36.9%) patients presented with two lymphoceles.

### 3.2. Classification of Lymphoceles and Characteristics

A total of 89 lymphoceles were radiologically detected in this patient population; 50/89 (56%) were right-sided lymphoceles, 34/89 (38%) were left-sided lymphoceles, while 5/89 (6%) of lymphoceles had a midline location.

**Figure 1:**
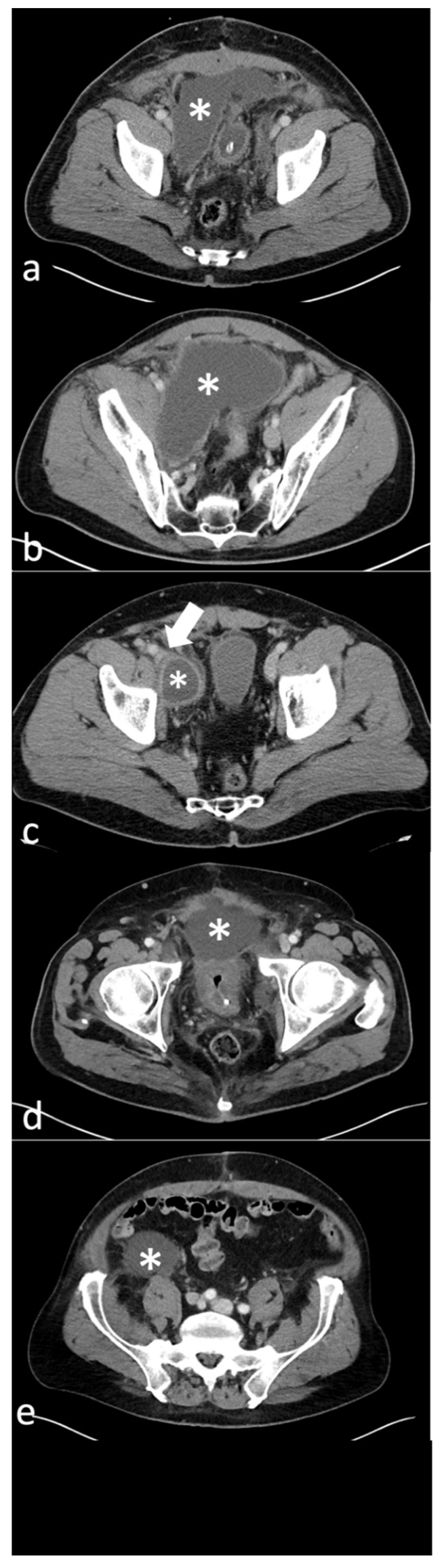
(**a**)-Axial CT image of a patient with a Type 1 lymphocele (*), 9 days after radical prostatectomy with pelvic lymph node dissection. (**b**)-Axial CT image of another patient with a large Type 2A lymphocele (*), 51 days after radical prostatectomy with pelvic lymph node dissection. (**c**)-Axial CT image of a different patient with a right-sided Type 2B lymphocele (*), 4.5 months after radical prostatectomy with pelvic lymph node dissection. The lymphocele is seen to compress the right external iliac vein (arrow) and shows peripheral enhancement with mild surrounding fat-stranding. (**d**)-Axial CT image of another patient with Type 3(*) + 2A lymphocele, 22 days after radical prostatectomy with pelvic lymph node dissection. (**e**)-Axial CT image of a further patient showing a right retroperitoneal component of a Type 4 lymphocele (*), 16 days after radical prostatectomy with pelvic lymph node dissection.

The detected 89 lymphoceles were classified on CT imaging in the following types according to the classification by Khoder et al. [7]: Type 1 (paravesical), Type 2A (lateral pelvic), Type 2B (deep pelvic), Type 3 (prevesical), Type 4 (pelvic with retroperitoneal extension) and Mixed (Table 1). Type 2A lymphoceles were the most common (48/89, 53.9%), followed by Type 2B (17/89, 19.1%) and Type 4 (15/89; 16.9%) (Table 1). CT images of the different types of lymphoceles are shown in Figure 1.

Fifty-six out of 65 patients (86.2%) underwent a single CT-guided intervention. Due to recurrence of lymphoceles following first intervention, 6/65 patients (9.2%) underwent two CT-guided interventions, and 3/65 patients (4.6%) underwent three CT-guided interventions. In six patients undergoing a second intervention, lymphocele recurrence was at the same location as the initially drained lymphocele, while in the other three patients it was due to small lymphoceles at a different location having increased in size. In the three patients undergoing a third intervention, lymphocele recurrence was at the same location as the originally drained lymphocele (at first and second intervention).

The maximum diameter of the detected lymphoceles (measured in the axial or coronal plane) ranged between 3.5 and 29 cm prior to the first intervention. The Shapiro–Wilk test did not show a normal distribution of values. The median maximum diameter was 8.5 cm (25%-; 75%-quartiles-7.0, 10.7). Thirty out of 65 patients (46%) showed a lymphocele with peripheral enhancement as a sign of potential infection, on a pre-interventional diagnostic contrast-enhanced CT.

The maximum diameter of recurrent lymphoceles after the first drain which required a second drain (total of nine lymphoceles) ranged between 3.7 cm and 8.6 cm. The Shapiro–Wilk test showed a normal distribution of values. The mean value was 7.9 cm (±2.1 cm). The maximum diameter of recurrent lymphoceles after the second drain which required a third drain (total of three lymphoceles) ranged between 3.6 cm and 5.7 cm with a mean value of 4.8 cm (±0.9 cm).

### 3.3. Interventions

**Figure 2:**
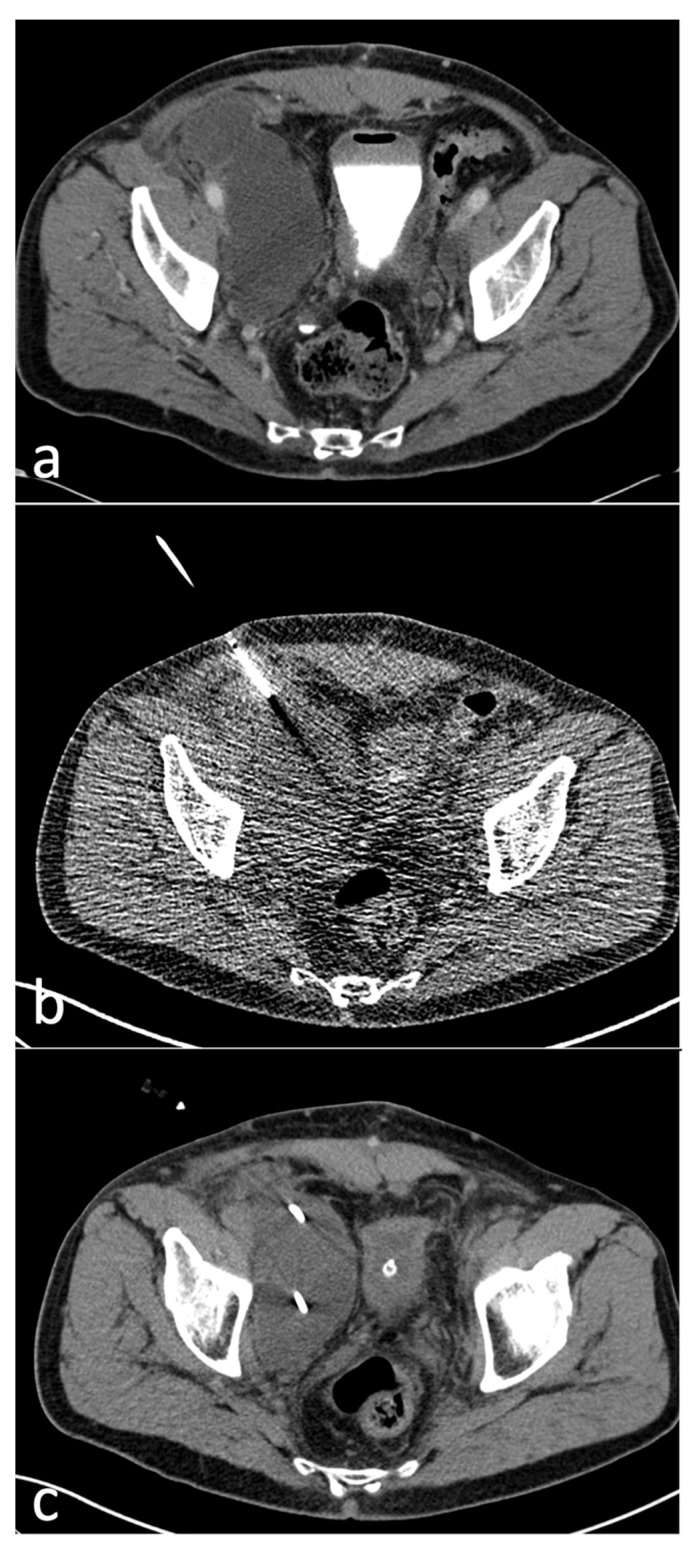
(**a**)-Axial CT image showing a right-sided Type 2A lymphocele, 10 days after radical prostatectomy and pelvic lymph node dissection. The maximum diameter of the lymphocele (cranial extension not shown on this image) was 12 cm. (**b**)-Axial CTF image showing 8F-drain insertion with the trocar technique in the same Type 2A lymphocele using a tube current-time product of 10 mAs. (**c**)-Axial post-interventional CT image of the same patient showing successful 8F-drain insertion into the lymphocele.

**Figure 3:**
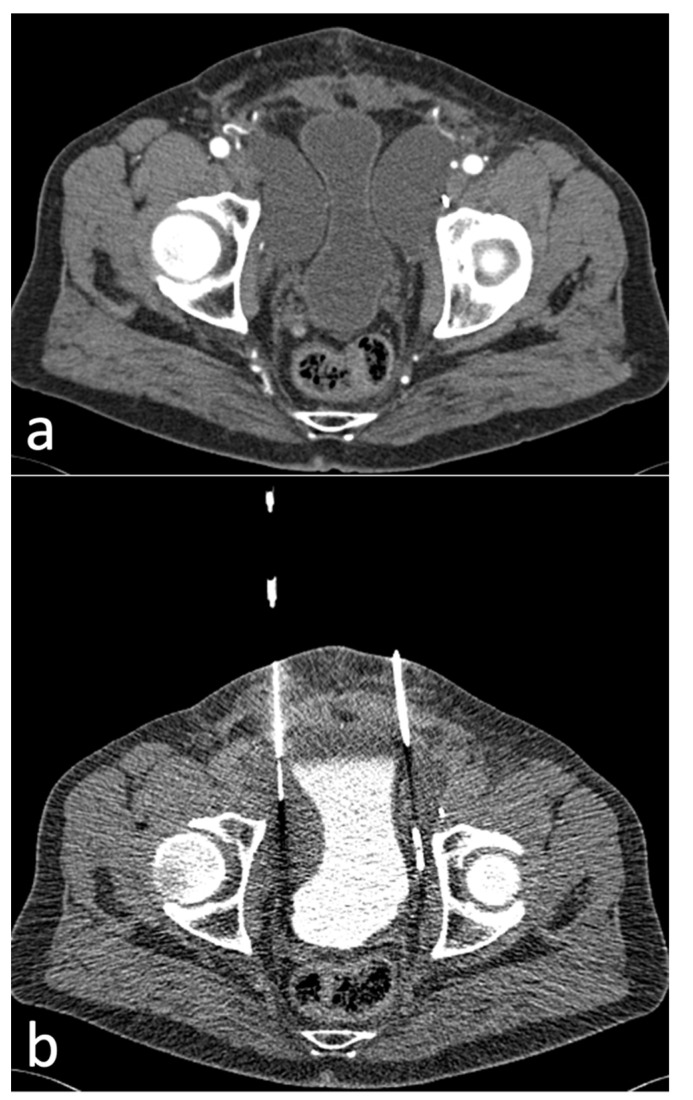
(**a**)-Axial CT image showing a different patient with bilateral Type 2A lymphoceles compressing the urinary bladder, 20 days after radical prostatectomy with pelvic lymph node dissection. (**b**)-Axial CT fluoroscopy image showing drainage insertion with the Seldinger technique in the right-sided Type 2A lymphocele using a tube current-time product of 25 mAs. An 8F pigtail drainage lies within the left-sided lymphocele.

A total of 77 CT-guided intervention sessions were performed in our cohort of 65 patients, with a total of 92 inserted drains. One drain was inserted in 62/77 (81%) sessions while two drains were inserted in 15/77 (19%) sessions. Eighty-nine out of 92 (97%) drains were placed using the curved trocar technique, while 3/92 (3%) were placed utilizing the Seldinger technique, both methods using intermittent quick-check CTF for guidance (Figure 2; Figure 3).

Single lumen drains used included Boston Scientific Flexima (8 to 12 French (F)) and Merit Resolve (7.5 to 12 F). In most cases (52/92; 57%), an 8F-drain was used. A 10-F drain was used in 29/92 (32%) of cases. 7-, 7.5- and 12-F drains were used in the remaining cases. 

### 3.4. Radiation Dose

The median DLP_pre_, DLP_intra_ and DLP_post_ (25%, 75% quartile) were 431 (271, 449) mGy*cm, 45 (19, 62) mGy*cm and 303 (194, 319) mGy*cm, respectively. The median total DLP was 723 (560, 871) mGy*cm. Table 2 shows the number of interventions performed for each increment of tube current-time product. 

### 3.5. Technical Outcome

CTF-guided lymphocele drain placement was technically successful in 100% of cases. A second CT-guided intervention was required at a later timepoint due to lymphocele recurrence in nine patients (one of these following drain dislocation in the ward), and a third intervention in three out of these nine patients (further details regarding second and third interventions above-Classification of Lymphoceles and Characteristics). 

On follow-up, 33/65 (50.1%) patients required a lymphocele fenestration, 32 of which underwent a laparoscopic marsupialisation, while one patient underwent an open surgical lymphocele fenestration.

### 3.6. Clinical Outcome

Clinical symptoms at the time of intervention included pain (47/65 patients; 72%), neuralgias (3/65 patients; 5%) and deep-vein thrombosis (4/65 patients; 6%). For all symptomatic patients with pain and neuralgias, a subjective improvement in symptoms was reported within the first 48 h after drain placement. 

Following the first CT-guided intervention and lymphocele drainage, patients remained in hospital for a mean period of 9 ± 5.9 days, with a minimum period of 2 days and a maximum period of 25 days after the intervention.

**Figure 4:**
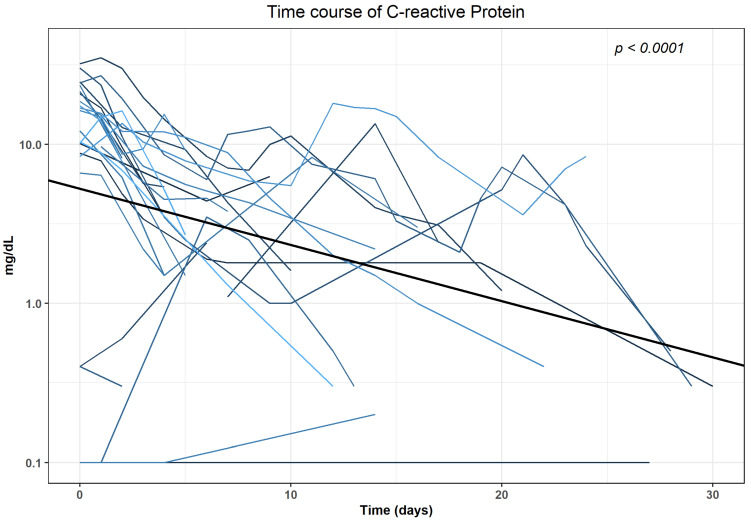
Time course of C-reactive protein for patients in the subgroup with no surgical intervention in the first 30 days after CTF-guided lymphocele drainage.

**Figure 5:**
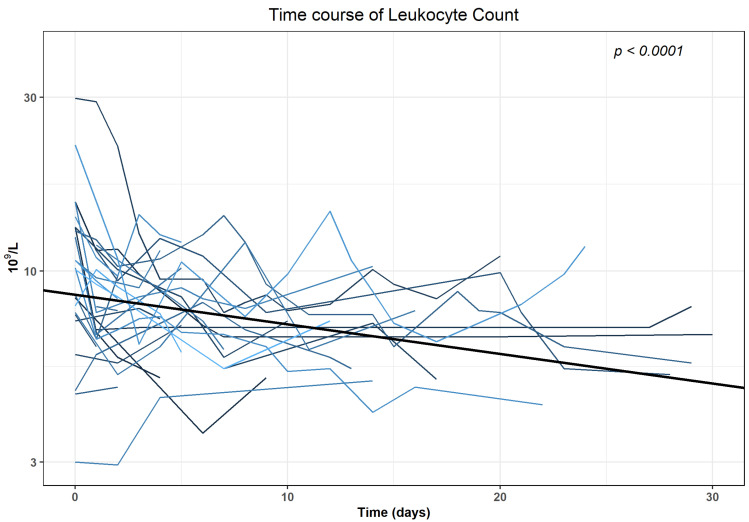
Time course of leucocytes for patients in the subgroup with no surgical intervention in the first 30 days after CTF-guided lymphocele drainage.

We analysed CRP and Leucocyte trends for 32 patients who did not undergo any surgical intervention in the 30 days following the first intervention. The median ((25%, 75% quartile)) CRP at the day of the intervention (baseline) was 14.3 (8.5, 21.4) mg/dL, Leucocytes were 10.2 (7.7; 13.2) × 10^9^/L. There were increased baseline levels of CRP (>0.5 mg/dL) in 18/32 patients (56.3%) and of Leucocytes (>9.8 × 10^9^/L) in 13/32 patients (40.6%). Time course of CRP and Leucocytes revealed a statistically significant (*p* < 0.0001) decrease within 30 days after intervention when analysed with generalised linear mixed models (GLMMs) in the subgroup of patients with no evidence of further surgical interventions in the first 30 days after intervention. The results of the final regression models are shown in Supplementary Table S1. The log-transformed values decreased with an average of −0.03541 mg/dL for CRP and −0.00812 × 10^9^/L for Leucocytes, respectively (Figure 4; Figure 5).

Clinical success as per our definition of decrease of initially elevated laboratory parameters was reached for CRP in 17 out of 18 interventions (94.4%) after (median (25%, 75% quartile)) 4 (3,5) days and for Leucocytes after 1 (1,3) day in 13 out of 13 cases (100%), respectively.

### 3.7. Complications

**Figure 6:**
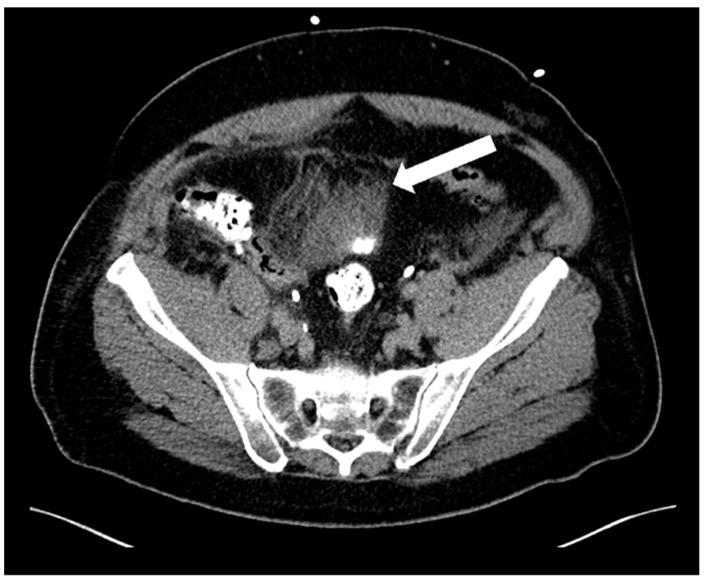
Axial CT image showing a small self-resolving haematoma (arrow) over the bladder dome as a complication of CT-guided drainage.

Adverse events (AEs) within 30 days related to CT-guided drainage consisted of only one minor complication (Grade 2 according to SIR)—a haematoma over the bladder dome (Figure 6) which resolved with conservative measures (watchful waiting).

## 4. Discussion

Pelvic LND, when performed during radical prostatectomy, increases morbidity in the treatment of prostate cancer (PCa), with lymphoceles being the most common adverse event (10.3% for extended lymph node dissection and 4.6% for limited lymph node dissection) [19]. While most lymphoceles are subclinical and rarely require intervention, complications such as infection or deep venous thrombosis secondary to pelvic vein compression can occur [3,7,11,20].

In our retrospective study including 65 patients with lymphoceles following radical prostatectomy and pelvic lymph node dissection, we achieved a technical success rate of CT-guided catheter drainage of lymphoceles of 100%. This is in agreement with another study which reported a high technical success rate in CT-guided catheter suction drainage of lymphoceles [21]. A particular feature in our study was the use of intermittent quick-check CT fluoroscopic acquisitions, using low-milliampere CTF at a tube current-exposure time product of 10–30 mAs, to reduce radiation dose to the patient and to the interventional radiologist. We achieved a median DLP of the sum of all intra-interventional CT fluoroscopic acquisitions of 45 mGy*cm. This value is lower than previously published data for the radiation dose for continuous CT-guided fluoroscopy for abdominal drainage (53 mGy*cm) [18]. Quick-check low-dose CT fluoroscopy enables safe placement of drains in pelvic lymphoceles due to their proximity to vascular structures and organs such as the bowel and bladder. Due to risk of injury to these structures, we favour the use of CT rather than US in guiding drain placement in pelvic lymphoceles, particularly for deeper lymphoceles. This approach was supported by our low complication rate in this patient cohort with no major complications and only one minor complication (haematoma over the bladder dome which resolved conservatively).

A further feature particular to our interventions was the use of the curved-trocar technique as described by Young et al. [16]. This allows for PD placement within the CT gantry under quick-check CT fluoroscopic acquisitions in most patients and is a safe and effective modification of the standard trocar technique. It facilitates CT-guided procedures hindered by CT gantry size limitations, particularly in patients with higher BMI [16,22]. The thin fluid-like consistency of lymphatic fluid within lymphoceles allowed us to use smaller 8F-drains in a large proportion of patients. 

For the patients who did not undergo further surgical intervention in the 30 days after CT-guided drainage, CRP and leucocytes showed a statistically significant decrease within 30 days after intervention. All symptomatic patients with pain and neuralgias reported a subjective improvement in symptoms within the first 48 h after drainage placement. Approximately half of the patients who underwent CT-guided catheter drainage needed to undergo further therapy with surgical fenestration, which is consistent with previously published data [6]. Our results demonstrate that CT fluoroscopy-guided catheter drainage provides an excellent technical and good clinical outcome.

An additional treatment option for lymphoceles is CT-guided drainage followed by sclerotherapy. The latter may be performed using various agents, including ethanol, povidone-iodine, tetracycline and doxycycline, bleomycin and fibrin glue [1]. Pooled data from a systematic review by Ten Hove et al. including 37 eligible studies (most involving gynaecologic oncologic surgery or renal transplantation with a few urological oncologic studies) with 732 lymphoceles showed more favourable success rates of percutaneous catheter drainage with sclerotherapy (proportion of successful interventions (0.872–0.890; 95% CI 0.710–0.948) when compared to percutaneous catheter drainage alone (0.612; 95% CI 0.490–0.722) in the treatment of postoperative lymphoceles in the pelvis [6]. Embolisation during lymphangiography showed the highest proportion of successful interventions 0.922 (95% CI: 0.731–0.981) [6]. A recent study including 35 patients with 39 symptomatic postoperative lymphoceles treated with vacuum-assisted suction drainage (inserted under CT guidance) showed a high clinical success rate with healing and total disappearance in 94.8% of symptomatic lymphoceles [21]. The authors reported a technical success rate of 100% and a complication rate of 4.6% (two minor complications—small haematoma in drainage canal; infection of the drainage canal) [21].

### Limitations

Our study is characterised by several limitations: First, this was a retrospective study with its inherent limitations, including a mixed patient spectrum from a large university hospital. Second, we only included patients who were referred to our department for CT fluoroscopy-guided drainage, the clinical outcome of patients primarily undergoing laparoscopic fenestration without prior CT fluoroscopy-guided drainage was not analysed in parallel. Third, our assessment of clinical outcome was limited to subjective symptom (pain) improvement within a 2-day post-interventional period, improvement in initially elevated laboratory values (CRP and leucocytes) over a 30-day period and a need for further surgical treatment with laparoscopic marsupialisation and did not include long-term patient follow-up as this was beyond the scope of our study. Additionally, results of microbiological (predominant bacteria) / cytological (differential seroma, liquified hematoma) analysis are not provided. Clinical outcome (e.g., QOL and pain intensity) was not quantified with a dedicated questionnaire (e.g., Visual Analogue Scale). No suction drainages were utilized and evaluated, only drainages with fluid passively following gravity. Drainage management on the ward was not evaluated (number of irrigations per day, etc.). No dedicated assessment of direct radiation exposure to interventional radiologists was performed. No analysis of procedure time and radiation exposure of CTF vs. sequential CT guidance was done.

## 5. Conclusions

In conclusion, our study demonstrates that, in patients with postoperative lymphoceles following radical prostatectomy and pelvic lymph node dissection, low-milliampere CT fluoroscopy-guided drainage is a safe treatment option with high technical success and good clinical success rates which can provide rapid symptom relief and may serve as definite treatment in a proportion of patients or as a bridging therapy prior to laparoscopic marsupialisation. An awareness of the low-milliampere CT fluoroscopy-guided technique is important as it is a very safe and effective alternative to US-guided drainage. Future studies may be performed to evaluate the benefits of suction vs. non-suction drainage and patient-related factors as predictors of long-term clinical outcome.

## Figures and Tables

**Table 1:** Classification of lymphoceles in the study cohort according to Khoder et al. [7].

Lymphocele Type	Description	Number	%
Type 1	Paravesical	2	2.2
Type 2A	Lateral pelvic	48	53.9
Type 2B	Deep Pelvic	17	19.1
Type 3	Prevesical	5	5.6
Type 4	Pelvic with retroperitoneal extension	15	16.9
Mixed	Mixed	2	2.2
Total		89	100

**Table 2:** Distribution of interventions for each tube current-time product.

mAs	No of Interventions	%
10	52/77	67.5
15	2/77	2.5
20	11/77	14.2
25	10/77	12.9
30	2/77	2.5

## Data Availability

The data presented in this study are available upon reasonable request from the corresponding author.

## Supplementary Materials

The following are available online at https://www.mdpi.com/article/10.3390/diagnostics12102394/s1, Supplementary Table S1: Parameters of the generalized linear mixed models (GLMMs) used in Figure 4; Figure 5.
