# Supplementary material for: Technical Outcome, Clinical Success, and Complications of Low-Milliampere Computed Tomography Fluoroscopy-Guided Drainage of Lymphoceles Following Radical Prostatectomy with Pelvic Lymph Node Dissection
Source: Diagnostics (Basel). 2022 Oct 1;12(10):2394. doi: 10.3390/diagnostics12102394 (PMC9600916; doi:10.3390/diagnostics12102394)
Supplement: Supplementary file 1 [file diagnostics-12-02394-s001.zip › diagnostics-1922618-supplementary.pdf]

Supplementary Table S1: Parameters of the generalized linear mixed models (GLMM) used in Figure 4 and 5.

	C-reactive Protein			Leukocyte Count		
Predictors	*Estimates*	*CI*	*p*	*Estimates*	*CI*	*P*
(Intercept)	0.72	0.51–0.93	**<0.001**	0.94	0.89–0.98	**<0.001**
timepoint day	-0.04	-0.04–-0.03	**<0.001**	-0.01	-0.01–-0.01	**<0.001**
**Random Effects**						
σ^2	0.12			0.01		
τ_{00}	0.30 Subject ID			0.01 Subject ID		
ICC	0.72			0.48		
N	31 Subject ID			32 Subject ID		
Observations	164			167		
Marginal R^2 / Conditional R^2	0.135 / 0.754			0.126 / 0.548		

CI: Confidence Interval; R2: Coefficient of Determination; σ2: distribution-specific variance; τ00: between-subject-variance; ICC: intraclass correlation coefficient, N: number of subjects
